# Regularity effect in prospective memory during aging

**DOI:** 10.3402/snp.v6.31238

**Published:** 2016-10-21

**Authors:** Geoffrey Blondelle, Mathieu Hainselin, Yannick Gounden, Laurent Heurley, Hélène Voisin, Olga Megalakaki, Estelle Bressous, Véronique Quaglino

**Affiliations:** CRP-CPO, EA 7273, Université de Picardie Jules Verne, Amiens, France

**Keywords:** regularity effect, age, lifespan, future intentions, episodic memory, planning, binding, spontaneous retrieval, multiprocess theory, clinical practice

## Abstract

**Background:**

Regularity effect can affect performance in prospective memory (PM), but little is known on the cognitive processes linked to this effect. Moreover, its impacts with regard to aging remain unknown. To our knowledge, this study is the first to examine regularity effect in PM in a lifespan perspective, with a sample of young, intermediate, and older adults.

**Objective and design:**

Our study examined the regularity effect in PM in three groups of participants: 28 young adults (18–30), 16 intermediate adults (40–55), and 25 older adults (65–80). The task, adapted from the *Virtual Week*, was designed to manipulate the regularity of the various activities of daily life that were to be recalled (regular repeated activities *vs*. irregular non-repeated activities). We examine the role of several cognitive functions including certain dimensions of executive functions (planning, inhibition, shifting, and binding), short-term memory, and retrospective episodic memory to identify those involved in PM, according to regularity and age.

**Results:**

A mixed-design ANOVA showed a main effect of task regularity and an interaction between age and regularity: an age-related difference in PM performances was found for irregular activities (older < young), but not for regular activities. All participants recalled more regular activities than irregular ones with no age effect. It appeared that recalling of regular activities only involved planning for both intermediate and older adults, while recalling of irregular ones were linked to planning, inhibition, short-term memory, binding, and retrospective episodic memory.

**Conclusion:**

Taken together, our data suggest that planning capacities seem to play a major role in remembering to perform intended actions with advancing age. Furthermore, the *age-PM-paradox* may be attenuated when the experimental design is adapted by implementing a familiar context through the use of activities of daily living. The clinical implications of regularity effect are discussed.

Prospective memory (PM) is to be distinguished from retrospective memory (i.e. the ability to remember past information). It refers to memory for actions to be performed in the future (Einstein & McDaniel, 1990). This ability is essential in everyday life to manage activities and is of upmost importance in maintaining independence and autonomy in old age. Classically, two components required to correctly perform delayed intentions have been identified: a prospective and a retrospective component. Prospective component refers to the remembering of an intended action to be performed at a specific time in the future (e.g. to take his/her insulin at 8 p.m.), whereas the retrospective component refers to the remembering of what needs to be done (e.g. to take his/her insulin). According to the nature of retrieval, Einstein and McDaniel (1990) have introduced time-based PM tasks, for which intention execution is auto-initiated by the person after a specific time interval (e.g. at 8 p.m.), and event-based tasks, for which intention execution is triggered by the appearance of an external event (e.g. a beeper sounds to remember to take insulin at 8 p.m.). PM seems to rely on more internal control and self-initiated processes than retrospective memory (Craik, 1986).

## PM: cognitive processes and tasks

McDaniel and Einstein (2000) suggest that executive resources need to be periodically allocated to retrieve intended actions in memory. The three main dimensions of executive functions (inhibition, shifting and updating; see Miyake et al., 2000) seems to be linked to PM performance (Schnitzspahn, Stahl, Zeintl, Kaller, & Kliegel, 2013). It is also applies to binding in working memory (Blondelle et al., 2015; Gonneaud et al., 2011; Hainselin et al., 2011), working memory (Cherry & LeCompte, 1999; West & Craik, 2001), processing speed (West & Craik, 2001; Zeintl, Kliegel, & Hofer, 2007), and metamemory (Meeks, Hicks, & Marsh, 2007; Meier, von Wartburg, Matter, Rothen, & Reber, 2011; Rummel, Kuhlmann, & Touron, 2013; Schnitzspahn, Zeintl, Jäger, & Kliegel, 2011; Smith, Souchay, & Moulin, 2011).

PM is assessed with two types of situations classically opposed in the literature: laboratory PM tasks and naturalistic ones. Laboratory PM tasks are computed-based, and participants need to perform a dual-task paradigm, such as standard PM task setting with a lexical-decision task (i.e. ongoing task) and a prospective task in which participants need to press a key when a word containing a specific syllable appears. Naturalistic PM tasks are performed in an ecological context, for instance during everyday life tasks (e.g. remember to call the experimenter at a specific time).

## PM in normal aging

Previous researches in normal aging have highlighted an intriguing pattern of age-related deficit in laboratory-based PM tasks while paradoxically, benefits are observed in naturalistic-based PM tasks (i.e. *Age-PM-Paradox*, see Rendell & Craik, 2000). This was also confirmed by a meta-analysis conducted by Henry, MacLeod, Phillips, and Crawford (2004). In laboratory-based PM tasks, young adults outperform older ones (Henry et al., 2004; Phillips, Henry, & Martin, 2008; Uttl, 2008), while the phenomenon is inverted (Rendell & Thomson, 1999; but see Will et al., 2009 with age-related cognitive impairment) or eliminated (Aberle, Rendell, Rose, McDaniel, & Kliegel, 2010) in more naturalistic-based PM tasks.

Considering the paradox, several authors tried to explain the inconsistent patterns of age-related difference in PM by referring to the multiprocess framework (Einstein & McDaniel, 2005; Einstein et al., 2005; McDaniel & Einstein, 2000) and Craik's aging memory theory (1986). Generally, the deleterious age-related effect in laboratory tasks could be attributed, at least in part, to a decrease in executive resources (Yuan & Raz, 2014 for meta-analysis) and self-initiated processes (Craik, 1986). The multiprocess viewpoint proposes that the presence or lack of age-related effect in PM retrieval is explained by relatively automatic retrieval processes (preserved during aging; e.g. Scullin, Bugg, McDaniel, & Einstein, 2011) or more strategic according to the configuration of the task. For instance, PM is posited to rely on automatic or spontaneous processes depending on certain conditions (Scullin, McDaniel, & Shelton, 2013). The most common automatic conditions are: (1) when few attentional resources are devoted to the ongoing task execution (Walter & Meier, 2014 for review), (2) when the processing required to carry out the ongoing task and the PM task are overlapped (e.g. if the ongoing task is to identify political figures during a naming test, a possible PM task would be to indicate when a politician wearing glasses appears) (Einstein, McDaniel, Richardson, Guynn, & Cunfer, 1995; Scullin, McDaniel, Shelton, & Lee, 2010), or (3) for salient PM cues (e.g. red glasses) (Rendell et al., 2011; Schnitzspahn, Horn, Bayen, & Kliegel, 2012; Smith, Hunt, McVay, & McConnell, 2007). When not into one of these three conditions, PM is supposed to be mainly supported by strategic and controlled processes, and performances thus decrease with aging.

Furthermore, other individual factors may reinforce the tendency to rely on spontaneous or controlled PM retrieval such as motivation (Moscovitch, 1982; Rendell & Craik, 2000) and personality factors (e.g. neurotic and conscientious people have better performance than perfectionism ones, see Cuttler & Graf, 2007). Age-related PM benefits observed in naturalistic setting can also be linked to higher frequency use of external aids, but also to better meta-cognitive knowledge by older adults (Schnitzspahn et al., 2011) or lifestyle (Rendell & Thomson, 1999). During aging, good planning performances are linked to good PM performances (Burgess, Veitch, De Lacy Costello, & Shallice, 2000; Martin, Kliegel, & McDaniel, 2003; Shallice & Burgess, 1991) and seem to avoid the need of strategic monitoring for PM cues retrieval (McDaniel & Einstein, 2000).

## Regularity effect in PM

Among the various factors influencing PM performances, the regularity effect has been sparsely investigated (Aberle et al., 2010; Rose, Rendell, McDaniel, Aberle, & Kliegel, 2010). Typically, PM task is considered as regular when performed on a daily basis and irregular when performed occasionally (Rose et al., 2010). Initial observations of this effect were made from journals or questionnaires in which participants were requested to note their PM failures. These observations highlighted that PM failures were reduced when the PM tasks were frequent and habitual (Andrzejewski, Moore, Corvette, & Herrmann, 1991). According to Van der Linden and Hupet (1994), recalling PM tasks is facilitated when it is achieved regularly rather irregularly, because such a recall could be guided by environment or by cues from previous activities. More recently, the regularity effect in PM was revealed in a study using the *Virtual Week* task paradigm (Rose et al., 2010). It showed that a regularity effect of PM tasks, and a strong interaction between task type and age: young and older adults, recalled more regular activities than irregular ones. Indeed, for regular activities, the age-related differences between young and older participants are strongly reduced or eliminated (Aberle et al., 2010). Adopting the multiprocess theory framework, McDaniel and Einstein (2000, 2007) proposed an account for these results. According to them, regular PM task retrieval may rely heavily on spontaneous retrieval mechanisms (e.g. in order to take insulin at dinner, the intention may spontaneously ‘pop into mind’ while having dinner). Hence, cognitive demand for these retrospective memory tasks is posited to be reduced, presumably because they are more frequently activated in memory (Rendell, Gray, Henry, & Tolan, 2007) than irregular activities requiring strategic monitoring for completion. The regularity effect was also tested in behavioral study using event-related potential and event-based PM tasks (Czernochowski, Horn, & Bayen, 2012). The results revealed higher monitoring frequencies for frequent than for rare PM cues, which suggest that this phenomenon is responsible for an increase of perceived importance for frequent PM cues.

It is noteworthy that most previous studies have taken into consideration a limited number of cognitive functions, which limits the scope of conclusions regarding cognitive processes involved in PM functioning in aging. Moreover, to our knowledge, no study has addressed in conjunction the links between regularity (i.e. whether everyday activities are regularly executed or not), PM and cognitive processes in a lifespan perspective. By ‘lifespan perspective’ we mean studying a sample of healthy young, intermediate, and older adults.

In line with the above considerations, the purpose of the present study was twofold. First, we aimed to explore and refine knowledge on aging and regularity effects in PM. Based on the present literature review, we predicted (1) a better recall for regular activities than for irregular ones, (2) an age-related difference on PM performances, and (3) an interaction between task regularity and age. To reach the first objective, we used an innovative method which consisted of checking the consistency between the regularity of each item attributed *a priori* by participants in order to approach, as much as possible, the real-life conditions.

Second, we assessed several cognitive functions such as planning, inhibition, shifting, short-term memory, binding in working memory, and retrospective memory in order to identify, in a broader lifespan perspective, the cognitive profiles linked to PM when taking into account regularity effect and age.

## Method

### Participants

A total of 69 participants were enrolled in our study (Table 1). They were divided into three groups: young (18–30), intermediate (40–55), and older (65–80). Younger participants were recruited from the undergraduate population of the University of Picardie Jules Verne. The other participants were recruited via flyers, were all leading active life, and were volunteers in the Amiens area. Informed consent form was obtained explaining about the objectives, justifications, and procedures of this investigation. Withdrawal from the study was possible at any time. Inclusion criteria were as follows: no neurological or psychiatric history, and a non-pathological score on the MMSE (>26/30) (Kalafat, Hugonot-Diener, & Poitrenaud, 2003), and Batterie d'Evaluation Cognitive (Signoret et al., 1989). The difference in the level of education observed in our sample is a commonly observed phenomenon in aging studies, including PM ones (Mioni, Stablum, Biernacki, & Rendell, 2015).

**Table 1:** Participants characteristics (standard deviations)

Variable	Young (*N=*28)	Intermediate (*N=*16)	Older (*N=*25)
Gender, women/men	13/15	7/9	12/13
Age in years, mean	23.98 (3.45)	47.89 (4.51)^a^	70.43 (6.27)^a^ ^b^
Level of education in years, mean	14.57 (1.89)	12.25 (2.70)^a^	10.20 (2.42)^a^

a Significant difference from the young adults

b significant difference from the intermediate adults.

The experimenters were master-level (MSc; graduated psychologists within the next months) students specifically trained to administer all the tests. Each participant was seen in a quiet room during 90 min.

### Material

**Fig. 1:**
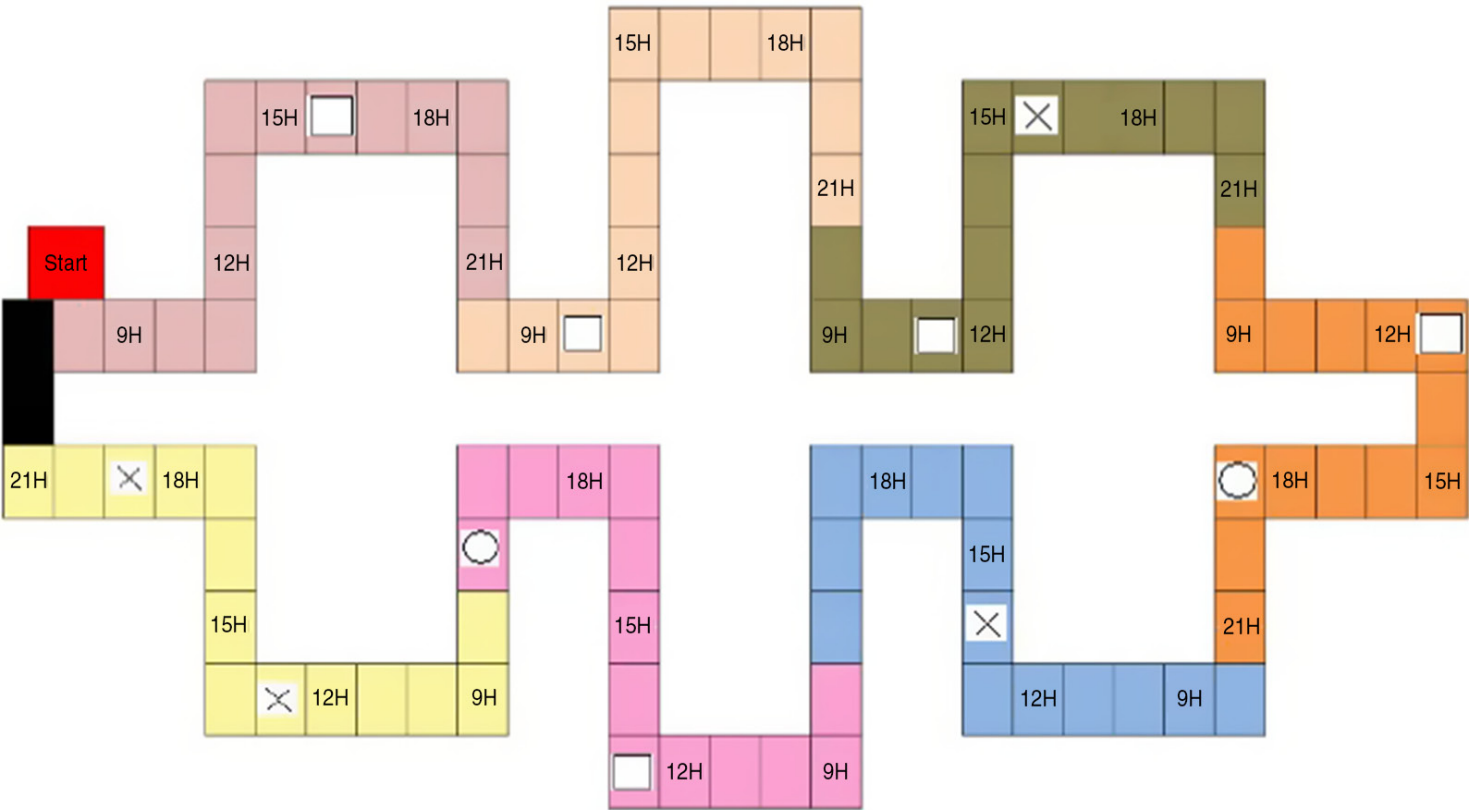
Prospective memory board game. The crosses, circles, and white squares are elements not discussed in this article because they are not related directly to the subject here.

A newly designed task inspired from the *Virtual Week* (Rendell & Craik, 2000) was used to assess regularity effect in PM. Participants had to perform throughout the week, a total of 34 tasks corresponding to activities of daily life. These were composed of 21 regular tasks (3 regular tasks each day, repeated 7 times in the week) and 13 irregular tasks (Monday to Saturday: 2 irregular per day; Sunday: 1 irregular). Tasks were semi-randomly distributed within the game board (see Fig. 1) to satisfy the regularity balance of each day depicted on the board. Each day (8 a.m. to 9 p.m.) was represented by a specific colored box on the board game.

A parallel version of our *Virtual Week* PM task was used in order to avoid any order effect for PM items. No differences were found between the two versions, *t*(47)=−0.07, *p*=0.94.

A pretest of the regularity of 65 activities of daily living was conducted on 60 participants (18–63 years) who were blind to the purpose of the research and were not part of the population sample of our experiment. For each item, participants were asked to rate regularity at the time of the post-evaluation on an 11-point Likert scale (0=*never*; 10=*every day*). We kept the 13 irregular and the 3 regular items corresponding to the most extreme mean values (i.e. <3 and >5, respectively; see Appendix 1).

### Procedure

**Fig. 2:**
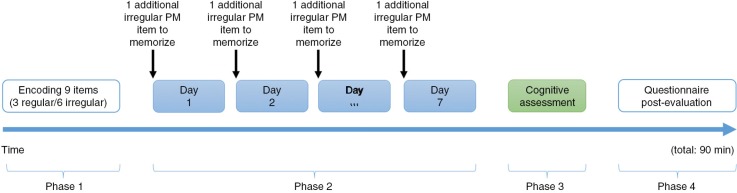
General procedure of the PM task.

The experiment included four phases. In the first phase, before the first die roll, the participants were asked to memorize nine tasks which were to be recalled during the second phase: three regular tasks to remember to do every day at the same time, and six irregular tasks to remember to do only one time at a specific moment. The second phase began with the experimenter announcing an additional task to perform at the appropriate time at the beginning of each day of the week (e.g. ‘It's Monday, and today, you must go to a medical appointment at 9 a.m.’). Then, using dice, the participants moved their pawn along the squares according to their score (e.g. move forward three squares when dice value is equal to three). Participants were requested to recall the activity orally when a pawn passed on a square which referred to a specific activity. One point was given for each activity correctly recalled, and the percentage of correct responses was computed. The PM task lasted approximately 15 min. In the third phase, participants performed the set of complementary cognitive tasks. During the fourth phase, participants were asked to rate the regularity of each activity on an 11-point Likert scale (0=*never*; 10=*every day*) (irregular: <3; regular: >5). The general procedure (see Fig. 2) and some data (i.e. cognitive processes scores) were similar to our previous study (Blondelle et al., 2015) in a lifespan perspective, emotional valence effect in PM, and the cognitive processes involved.

### Complementary cognitive tasks

We tested both executive and episodic memory functions after the PM task. Executive functioning was assessed by using the French versions of the Behavioral Assessment of the Dysexecutive Syndrome Zoo Map Test (BADS; Wilson, Alderman, Burgess, Emslie, & Evans, 1996), Stroop test (Stroop, 1935), and Trail Making Test (TMT; Reitan, 1958). Visuospatial short-term memory was also evaluated (Quinette et al., 2013) as well as binding processes (Quinette et al., 2013). Retrospective episodic memory assessment included the Logical Memory Test (Wechsler, 2001) and the French adaptation of the Free and Cued Selective Reminding Test (FCSRT; Buschke, 1984).

## Statistical method

Statistical analysis was performed using JASP 0.7.5.5 (JASP Team, 2016). Effect sizes were measured by omega squared (ω^2^). The corrected effect size omega squared was conceived as an alternative to eta squared that estimates the amount of variance explained by the entire population, and not only on the sample (Lakens, 2013). We conducted a 3×2 mixed-design omnibus ANOVA on mean percentages of activities correctly recalled with task regularity (regular and irregular) as a within-subject factor and age groups (young, intermediate, and older) as a between-subject factor and Tukey's honestly significant difference (HSD) analyses for *post hoc* pairwise comparisons. One-way ANOVAs were performed on the mean percentage of total PM scores (regular+irregular) and the average scores obtained in the complementary cognitive tests to explore their impacts with regard to aging. Spearman's correlations between PM indicators and the cognitive assessment measures were conducted for the whole sample to identify the patterns of cognitive processes involved according to task regularity. Partial correlations (by controlling age factor) were also computed by taking into account age group to determine if they were mainly due to age or not. Holm-Bonferroni's correction was used to avoid type I errors (Gaetano, 2013; Holm, 1979). This consisted of correcting *p*-values according to the total number of comparisons performed (i.e. here correlations) and *p*-value ranks. For all analyses, the rejection level for inferring statistical significance was set at *p*<0.05.

**Fig. 3:**
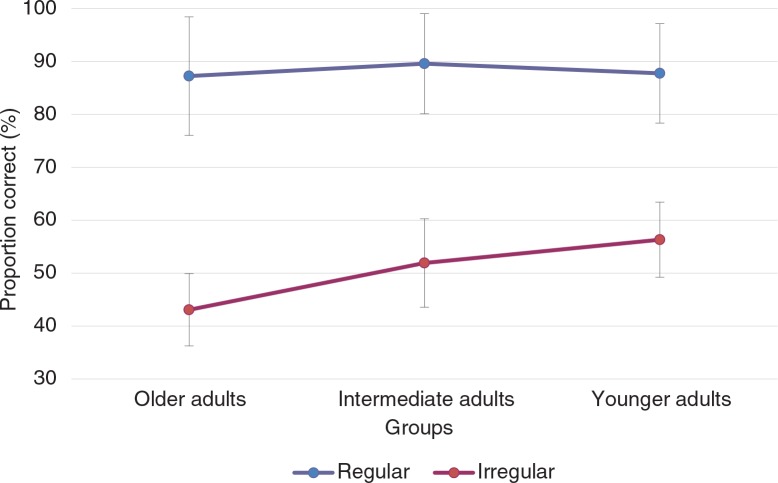
Mean proportion correct for regular (repeated) and irregular (non-repeated) PM activities across age groups.

**Fig. 4:**
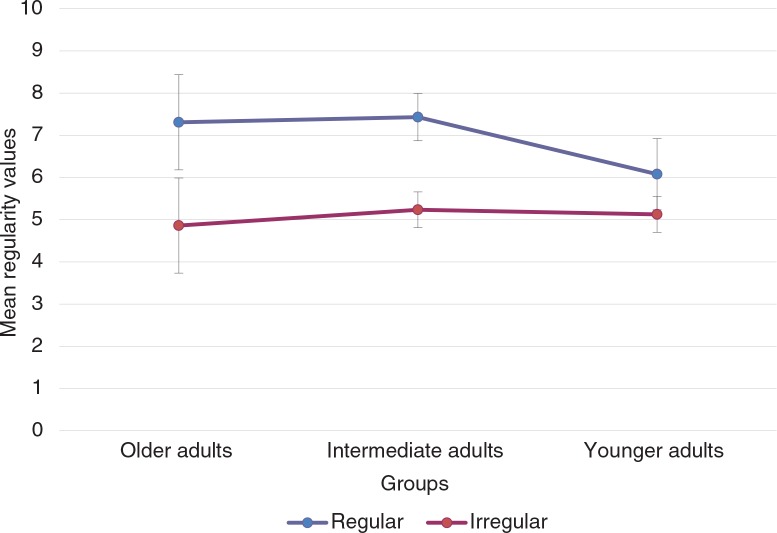
Mean of regularity given after completing PM test for regular (repeated) and irregular (non-repeated) PM activities.

Mean proportions for correctly recalled regular and irregular activities and total PM score on the PM task are presented in Fig. 3. Mean regularity given by participants for prospective items is set out in Fig. 4.

### Prospective memory

A 3 (Age groups [young, intermediate, older])×2 (Regularity of the activities [regular, irregular]) mixed ANOVA revealed a significant main effect of regularity [*F*(1, 66)=291.87; MSE=159.00, *p*<0.001, *ω*^2^=0.80], where recall of irregular activities was lower than regular ones. The interaction between age groups and regularity was also significant [*F*(2, 66)=3.36; MSE=159.00, *p*<0.05, *ω*^2^=0.01]. Tukey's HSD showed that older adults had poorer PM performance for irregular activities than young adults. All other comparisons were no significant. Likewise, no effect of age group was observed on the total PM scores [*F*(2, 66)=1.45; MSE=463.80, *p*=0.24, *ω*^2^=0.01].

Analyses were also performed on participants’ regularity evaluation scores collected after the PM task. A 3 (Age groups [young, intermediate, old])×2 (Post-evaluation of regularity of the activities [regular, irregular]) mixed ANOVA revealed a main effect of post-evaluation of regularity [*F*(1, 66)=74.46; MSE=1.47, *p*<0.001, *ω*^2^=0.49], where irregular activities were evaluated lower than regular ones, and a significant interaction between age groups and post-evaluation of regularity [*F*(2, 66)=5.24; MSE=1.47, *p*<0.05, *ω*^2^=0.06]. However, Tukey's HSD analyses for pairwise comparisons between regular and irregular activities and between age groups were no significant. No effect of age groups was found [*F*(2, 66)=1.94; MSE=1.47, *ns*].

### Cognitive assessment

Detailed scores for cognitive assessment are shown in Table 2. One-way ANOVAs showed an age group effect on planning [*F*(2, 66)=4.91; MSE=106,698, *p*=0.01, *ω*^ 2^=0.10], inhibition [*F*(2, 66)=9.78; MSE=0.62, *p*<0.001, *ω*^2^=0.20], shifting [*F*(2, 66)=13.40; MSE=906.30, *p*<0.001, *ω*^2^=0.26], both verbal and visuospatial span [*F*(2, 66)=5.17; MSE=1.19, *p*<0.05, *ω*^2^=0.11, and *F*(2, 66)=12.65; MSE=1.16, *p*<0.001, *ω*^2^=0.26], binding [*F*(2, 66)=15.70; MSE=7.93, *p*<0.001, *ω*^2^=0.30], and retrospective episodic memory [*F*(2, 66)=12.59; MSE=2.58, *p*<0.001, *ω*^2^=0.25]. Overall, Tukey's HSD showed that participants in the older group had lower performance for FCSRT and produced higher time responses in the Stroop test (interference condition) for than others.

**Table 2:** Cognitive assessment for young, intermediate, and older groups (standard deviations)

Variable	Young	Intermediate	Older
Zoo Map Test: sequencing score	6.61 (2.42)	5.25 (2.86)	7.04 (1.99)
Stroop interference: errors	0.12 (0.44)	0.06 (0.25)	0.50 (2.00)^a^
TMT B: errors	0.07 (0.26)	0.31 (0.60)	0.04 (0.20)
Visuospatial span	5.79 (1.32)	4.81 (0.66)^a^	4.32 (0.99)^a^
Binding: processing	−0.11 (0.07)	−0.21 (0.09)^a^	−0.24 (0.12)^a^
FCSRT: delayed free recall	14.57 (1.37)	13.69 (2.12)	12.36 (1.36)^a^ ^b^

a Significant difference from the young adults

b significant difference from the intermediate adults.

BADS, Behavioral Assessment of the Dysexecutive Syndrome; TMT, Trail Making Test; FCSRT, Free and Cued Selective Reminding Test.

### Correlations (r_s_ values) between cognitive assessment and regularity

All correlation coefficients are given in Table 3. Both regularity measures were linked to planning. In addition, inhibition, visuospatial short-term memory, binding, and retrospective memory measures were specifically correlated with irregular activity scores, but not shifting. Finally, correlations were no significant between regular activity scores and inhibition, shifting, visuospatial short-term memory, binding, and retrospective memory.

**Table 3:** Correlation coefficients (*r*_*s*_ values) between cognitive performances and both regular and irregular activities

Variable	Regular	Irregular
Zoo Map Test—BADS: sequencing score	0.35*	0.28*
Stroop interference: errors	−0.18	−0.58**
TMT B: errors	0.17	0.17
Visuospatial span	0.14	0.34*
Binding: processing	0.15	0.48**
FCSRT: delayed free recall	0.14	0.32*

* *p*<0.05

** *p*<0.01.

BADS, Behavioral Assessment of the Dysexecutive Syndrome; TMT, Trail Making Test; FCSRT, Free and Cued Selective Reminding Test.

### Partial correlations (r_s_ values) between regularity and cognitive assessment, when taking into account age groups

Spearman partial correlations between regularity and cognitive assessment for the three age groups are set out in Table 4.

**Table 4:** Correlation coefficients (*r*_*s*_ values) between age groups, cognitive performances, and PM tasks scores for both regular and irregular activities

Variable	Regular			Irregular		
	
	Young	Intermediate	Older	Young	Intermediate	Older
Zoo Map Test—BADS: sequencing score	0.20	0.52*	0.59**	0.09	0.42*	0.33*
Stroop interference: errors	0.04	0.10	−0.10	−0.23	−0.31	−0.44
TMT B: errors	0.17	−0.16	0.22	0.20	0.03	−0.09
Visuospatial span	−0.03	0.24	0.25	−0.07	0.11	0.34
Binding: processing	0.35	0.22	0.05	0.28‡	0.56*	0.29*
FCSRT: delayed free recall	0.24	−0.36	0.06	0.08	0.02	0.12

* *p*<0.05;

** *p*<0.01

‡ approached significance (*p*=0.06).

BADS, Behavioral Assessment of the Dysexecutive Syndrome; TMT, Trail Making Test; FCSRT, Free and Cued Selective Reminding Test.

For young participants, no correlation was significant regarding both regular and irregular activity scores. For intermediate participants, both regular and irregular activity scores were linked to planning, and this also applies to binding only for the irregular condition. For older participants, both regular and irregular activity scores were correlated with planning, which is also the case for processing scores on the binding task in the irregular condition. No correlation of the PM measure was significantly correlated with shifting and retrospective memory.

## Discussion

We are the first to demonstrate that distinct cognitive profiles are involved in PM according to both regularity and age. Results showed a main effect of regularity. Here we discuss regularity, age, cognitive profiles, and the clinical implications of these findings.

The first aim of this study was to assess age, regularity effect, and interaction in PM. We tested the hypothesis that regular activities were more likely to be recalled than irregular ones. The findings revealed a better recall when activities were regular than irregular. This is in line with a previous study using the *Virtual Week* (Rose et al., 2010) and the theoretical proposal provided by the multiprocess theory (McDaniel & Einstein, 2000, 2007). One possible explanation for this pattern of results may be the decrease of cognitive load for regular tasks (Rendell et al., 2007). First, regular activities were repeatedly recalled every day at the same time throughout the test. On the opposite, irregular activities were recalled only once. Second, the repetitiveness of regular activities might have helped strengthening the moment when (prospective component) and what (retrospective component) had to be recalled. Better performances for regular (3 associations for 21 items) compared to irregular (13 associations for 13 items) activities could be explained by the number of total time–action (prospective and retrospective components) associations to be encoded. Having three associations to remember could be easier than 13, and the repetitiveness of the three regular associations could reinforce memory for action. In a fundamental perspective, it would be worthwhile to evaluate separately both prospective and retrospective components to find out if a variation of PM performance is observed between regular and irregular tasks. As part of these analyzes, it should be taken into account irregular activities announced at the beginning of the test and those announced during the test.

In addition to the classic regularity effect observed, we found an age-related difference for irregular activities. Recall of irregular activities was lower for older adults’ than for younger ones. This is because older adults’ difficulties in PM are more pronounced for conditions that are more demanding in terms of monitoring processes to maintain the intention in memory. This is likely the case with irregular activities, in line with previous findings (Rose et al., 2010) and consistently with the multiprocess theory.

Our results also highlighted that mean percentages of regular activities correctly recalled were higher than irregular ones. In post-evaluation condition, mean regularity values for regular activities were also higher than irregular ones. This pattern of results shows the consistency of recall with post-evaluation measures. It also highlights the usefulness of our method in ensuring that activities considered as regular and irregular in the present experiment are perceived as such by each participant with regard to his/her everyday life.

The present results allow some conclusions about the *age-PM-paradox* with a particular emphasis on the classically deleterious age effect observed in PM for laboratory tasks. No age-related difference was observed on the total PM scores between the three groups. This result notably goes against other studies that have shown a deleterious effect of aging in standard laboratory-based PM tasks (Uttl, 2008, for meta-analysis). In the *age-PM-paradox* perspective (Henry et al., 2004), the relative inconsistency of our results regarding the literature might be explained by two dimensions of ecological validity raised by Phillips et al. (2008): the task familiarity (familiar or novel) and the nature of the task occurrence (naturally occurring or artificial). Although we used laboratory-based tasks (i.e. artificial tasks: laboratory tasks put in place by the experimenter, as defined by Phillips et al.'s classification), we implemented a familiar context through the use of activities of daily living. This latter aspect may be one explanatory factor of this result. Consistent with our results, Garden, Phillips, and MacPherson (2001), using a planning task in a context of ecological assessment (i.e. shopping errand task), did not highlight any age effect between participants. In our experiment, planning was linked to PM performances and could be a factor that may explain the different results with regard to other studies (Kliegel, Eschen, & Thöne-Otto, 2004). Otherwise, Azzopardi, Juhel, and Auffray (2015) have shown that age effect could not predict PM performances alone, except when the interaction with both age and executive functioning factors was considered. All these results suggest that the *age-PM-paradox* might just be a material/paradigm effect. By adapting experimental designs and taking into account cognitive processes, this paradox could definitely disappear.

In the present study, results also suggest that age effect would not be present in our lifespan perspective (young, intermediate, and older adults), but can possibly emerge when young adults (<30 year-old participants) are compared to old adults (aged 70 years and above) as reported by Kvavilashvili, Cockburn, and Kornbrot (2012). In order to neatly access the *age-PM-paradox*, we recommend to split the old group into (at least) two subgroups of young–old and old–old participants, as many studies in the field have done (e.g. Kliegel & Jäger, 2006; Mioni et al., 2015; Rendell & Thomson, 1999).

The second objective was to explore, in a broader lifespan perspective, the nature of cognitive profiles linked to PM when taking into account regularity effect and age.

For regular activities’ performances, we found a unique significant correlation with the Zoo Map Test sequencing scores. Furthermore, correlations were also significant for both regular and irregular activities’ performances when age groups are considered, except for the younger adults, and thus, generally showed similar cognitive profiles between the both intermediate and older adults regarding the two measures of PM. This pattern of results suggests that planning plays a major role for remembering to perform intended actions (Kliegel et al., 2004; Martin et al., 2003; Niedźwieńska, Janik, & Jarczyńska, 2013) and clarifies its involvement within our event-based PM task in the middle-age (48 years on average). It also supports the proposal of McDaniel and Einstein (2000). They proposed that type and degree of planning can affect the extent of PM performances, which is underpinned by relatively more automatic retrieval processes and lower levels of cognitive resources demands. These results lead us to suggest that it is important for future research to investigate the role of planning in PM in a longitudinal study of adults covering a wide age range (e.g. Kliegel, Mackinlay, & Jäger, 2008a), by adopting a more cognitive profile-centered approach. Finally, no significant correlations were found between regular activities and delayed free recall in the FCSRT. This last result is not surprising. It may be due to the minimal demand on retrospective memory for repeated regular activities. The regular occurrence of a task could allow the person to periodically obtain cues that could facilitate the intention retrieval in memory.

In addition to the significant correlations found between the Zoo Map Test sequencing scores for the two measures of regularity, irregular activities’ performances were also significantly correlated to errors produced in the Stroop test, visuospatial span, processing scores on the binding task, and delayed free recall for the FCSRT. Moreover, involvement of binding for both intermediate and older adults suggests the importance of multimodal information integrations processes in binding target with the intention. These processes are known to be sensitive to aging in retrospective episodic memory (Naveh-Benjamin, 2000). We can assume that age-related difficulties for irregular activities’ recall in PM could be associated with a binding impairment which would occur at the middle-age itself. Finally, the unique and non-significant relationships between irregular activities and errors produced in the TMT, even when age groups are considered, are consistent with two studies revealing that both time- and event-based PM tasks seem to rely on different executive processes (Gonneaud et al., 2011; Kliegel, Ramuschkat, & Martin, 2003). For example, Kliegel et al. (2003) found that shifting was required for time-based PM tasks executions, while event-based PM tasks involved inhibition processes to avoid any distraction from irrelevant items. It is likely that the more PM task involves inhibition, binding, and both short-term and episodic retrospective memory processes, the more it is sensitive to aging. This assumption is supported by (1) the lower levels of cognitive performance for older adults compared to the other age groups and, (2) the lower recall for irregular activities in the old group rather than younger.

According to the multiprocess framework, we can assume that retrieval mechanisms for both regular and irregular activities are supported by two different pathways. First, the better recall for regular activities for each age group is probably underpinned by more spontaneous retrieval processes (bottom-up), well preserved during aging in PM (Mullet et al., 2013; Scullin et al., 2011). Second, irregular activities seem to rely more on self-initiated processes (top-down), which are sensitive to age (Craik, 1986). These processes maintain the intention and monitor the environment to trigger the retrieval of the intention (McDaniel, Umanath, Einstein, & Waldum, 2015) and involve the frontoparietal network (Cona, Scarpazza, Sartori, Moscovitch, & Bisiacchi, 2015).

## Clinical implications

The ability to successfully remember to do an action in the future is essential to pursue an autonomous life (Kliegel, Mackinlay, & Jäger, 2008b). Considering the importance of day-to-day PM situations, the negative consequences of its impairment can cause both personal and professional difficulties. Specifically, forgetting to perform an action that one has planned to do, for example, buy milk on the way home, turn off the oven, take medication, pay bill, or go to an appointment, can be encountered by anyone. However, such forgetting tends to increase over 70 years old (Kvavilashvili, Kornbrot, Mash, Cockburn, & Milne, 2009), with a decline of PM abilities even starting from 40 to 50 years (Mäntylä & Nilsson, 1997). In the light of our findings, several clinical ways for neuropsychologists to take care of patients presenting PM can be devised with a particular emphasis on regularity action aspects. Considering the regularity effect observed, clinicians should encourage patients with PM difficulties in reinforcing their habits with more focus on action planning for both regular and irregular actions to elaborate their intentions. This issue is particularly interesting with regard to Kliegel, Martin, McDaniel, Einstein, and Moor's (2007) findings highlighting that older adults may have better planning abilities when they are guided toward an effective structuring action plan. Moreover, the similar cognitive profiles between both intermediate and older adults alert us on the need of an earlier diagnosis and management of PM difficulties. Indeed, it may be useful to agree on the routinization of some irregular activities to improve both intermediate and older adults’ PM. These adaptive strategies are already part of several management programs. For instance, routines are very often recommended for patients with dementia (e.g. Bergua & Bouisson, 2008). Indeed, repetitive actions that are always performed at the same time are rarely forgotten. In the same way, an object always in the place is readily found, probably because it is supported by a cue-driven spontaneous retrieval processes, which seems preserved in aging (Scullin et al., 2011). Taken together, these findings require us to consider the importance of planning strategies (taking into account environmental aspects) and routinization, combined with careful observation in everyday life through an individual assessment of patients to reliably appreciate patients’ PM abilities.

In conclusion, the present study showed that task regularity is a determining factor that could explain age-related variability in PM performances across age. As proposed by the multiprocess framework, age differences are eliminated for regular activities’ recall. Moreover, our data support the idea that age-related difference for irregular activities’ recall may be associated with decrease of several executive processes. Our data also suggest that the *age-PM-paradox* may be undermined when the experimental design is adapted by implementing a familiar context through the use of activities of daily living. Routinization and planning of important irregular activities may be a possible measure in caring for patients with PM difficulties. Further research should address this issue.

## Appendix

**Appendix 1:** Activities included in the PM task

Activities	Time	Regularity
Having breakfast	8 a.m.	Regular
Going to the bakery	5 p.m.	Regular
Taking medication	8 p.m.	Regular
Watching television	9 p.m.	Irregular
Taking out the trash	4 p.m.	Irregular
Taking an appointment at the dentist	10 a.m.	Irregular
Going to the restaurant	6 p.m.	Irregular
Listening to music	4 p.m.	Irregular
Going to the post office	10 a.m.	Irregular
Going to the doctor	1 p.m.	Irregular
Using the computer	6 p.m.	Irregular
Going to the cinema	1 p.m.	Irregular
Going to the bank	2 p.m.	Irregular
Mowing the lawn	1 p.m.	Irregular
Visiting a friend	2 p.m.	Irregular
Gardening	3 p.m.	Irregular
